# FOSL1 promotes tumor growth and invasion in ameloblastoma

**DOI:** 10.3389/fonc.2022.900108

**Published:** 2022-09-15

**Authors:** Gan Xiong, Shengqi Ouyang, Nan Xie, Jiaxiang Xie, Wenjin Wang, Chen Yi, Ming Zhang, Xiuyun Xu, Demeng Chen, Cheng Wang

**Affiliations:** ^1^ Hospital of Stomatology, Sun Yat-sen University, Guangzhou, China; ^2^ Guangdong Provincial Key Laboratory of Stomatology, Sun Yatsen University, Guangzhou, China; ^3^ Guanghua School of Stomatology, Sun Yat-sen University, Guangzhou, China; ^4^ Center for Translational Medicine, The First Affiliated Hospital, Sun Yat-sen University, Guangzhou, China

**Keywords:** ameloblastoma, FOSL1, kinetochore metaphase signaling, epithelial-mesenchymal transition pathway, cell proliferation

## Abstract

**Background:**

FOSL1, a key component of the Activating protein-1 (AP-1) transcriptional complex, plays an important role in cancer cell migration, invasion, and proliferation. However, the impact of FOSL1 in ameloblastoma (AM) has not been clarified. Herein, we aimed to assess the expression of FOSL1 and investigate its functional role in AM.

**Methods:**

The expression of FOSL1 was examined based on an immunohistochemistry analysis of 96 AM samples. Cell proliferation, migration, invasion, and tumorigenesis were assessed using Cell Counting Kit-8 (CCK-8), colony formation, Transwell, and sphere formation assays. RNA sequencing (RNA-seq) was employed to investigate the molecular alterations of AM cells upon FOSL depletion. Microarrays of AMs were downloaded from the Gene Expression Omnibus (GEO) database for bioinformatics analysis. In addition, patient-derived AM organoids were used to evaluate the therapeutic value of the AP-1 inhibitor.

**Results:**

FOSL1 was detected in the nuclei of AMs and upregulated in conventional AMs compared to unicystic AMs and normal oral epithelium. Compared with primary AM, FOSL1 expression was significantly increased in recurrent AM. Genetic knockdown of FOSL1 suppressed the proliferation, migration, invasion, and sphere formation of AMs. Similar results were also observed by pharmacological inhibition of AP-1 activity. Moreover, the AP-1 inhibitor T5224 impeded the growth of organoids derived from AM patients. Mechanistically, our Ingenuity Pathway Analysis (IPA) and gene set enrichment analysis (GSEA) results revealed that depletion of FOSL1 inactivated kinetochore metaphase signaling and the epithelial–mesenchymal transition pathway and then impaired the aggressiveness of AM cells accordingly.

**Conclusion:**

FOSL1 promotes tumor recurrence and invasive growth in AM by modulating kinetochore metaphase signaling and the epithelial–mesenchymal transition pathway; thus, it represents a promising therapeutic target for AM treatment.

## Introduction

Ameloblastoma (AM) is a locally aggressive benign odontogenic epithelial tumor that usually occurs in the jawbone and arises from the dental lamina, epithelial rests of Malassez, or the basal cell layer of the oral epithelium (1, 2). In 2017, the WHO’s new classification of AM was simplified and made more practical, and it reserved two subtypes (unicystic type and extraosseous/peripheral type) and referred to the remaining types as AMs (conventional) (3). Meanwhile, the new classification defines metastasizing AM as a benign tumor and simplifies the classification of ameloblastic carcinoma, which is of great importance for clinical diagnosis and treatment. Clinically, AM displays an aggressive behavior with marked local invasion and bone resorption, which leads to tooth loss, malocclusion, paresthesia, pain, limited mouth opening, facial deformity, and airway obstruction (3). Currently, the management of AM is based on surgery, and recurrence is still a major challenge (4). A lower recurrence rate might be achieved by radical surgery, which always results in esthetic and functional problems (5, 6). Thus, it is urgent to elucidate the molecular mechanisms that govern the aggressiveness of AM and then identify a therapeutic target for AM management.

FOSL1, a key component of the AP-1 transcriptional complex, has been confirmed to be involved in many biological processes, including the cell cycle, migration, invasion, proliferation, and cell plasticity (7, 8). Interestingly, several studies have shown that FOSL1 functions as a novel osteoclastogenic protein that can modulate the activity of osteoclasts (9–11). Moreover, increasing evidence confirms that FOSL1 is highly expressed in invasive cancers and can enhance cell migration, proliferation, and stemness in several solid cancers, including lung cancer, pancreatic cancer, breast cancer, cholangiocarcinoma, and head and neck squamous cell carcinoma (12–15). These findings indicate that FOSL1 might be a novel target for simultaneously preventing tumor progression and bone destruction. However, the functional role and expression of FOSL1 in AM have not been documented.

In this study, we found that FOSL1 can be detected in AM, which is markedly increased in conventional AM compared to unicystic AM. Functional studies showed that depletion of FOSL1 inhibited cell proliferation, migration, invasion, and stemness in AMs. Mechanistically, kinetochore metaphase signaling and the epithelial–mesenchymal transition (EMT) pathway were significantly suppressed in AM cells upon FOSL1 depletion. Taken together, these findings indicate that increased expression of FOSL1 enhances the aggressiveness of AM by modulating the cell cycle and EMT, which might serve as a novel target for AM treatment.

## Materials and methods

### Human ameloblastoma samples

In this study, 96 AM samples were obtained from the Department of Oral Pathology, Guanghua School of Stomatology, Sun Yat-sen University. Tumor samples were treated in accordance with the guidelines of the Declaration of Helsinki. This study was approved by the Medical Ethics Committee of the Hospital of Stomatology Sun Yat-Sen University (KQEC-2022-35-01).

### Immunohistochemistry

Immunostaining was performed using a rabbit two-step immunohistochemistry kit (Zsbio, PV6001). Briefly, the samples were fixed with 4% paraformaldehyde, embedded in paraffin, and sectioned at a thickness of 4 μm. The sections were then baked for 1 h at 65°C, deparaffinized in an alcohol series, and subjected to heat-induced epitope recovery (Zsbio, ZLI-9065). Subsequently, the sections were incubated with FOSL1 antibodies (Abcam, AB124722) and secondary biotinylated antibody (Zsbio, PV-6001-18). In this study, 3,3’-diaminobenzidine (DAB) was used to detect the immunoreactions. The immunohistochemistry (IHC) score of each sample was assessed by the staining intensity and the percentage of positive cells, and it was evaluated by the following formula: 1 × percentage of cells staining weakly + 2 × percentage of cells staining moderately + 3 × percentage of cells staining strongly.

### Cell lines, siRNA transfection, and treatment

The hTERT^+^-AM cell line was an immortalized cell line provided as a gift from Prof. Qian Tao (16). All cells were cultured in Dulbecco's Modified Eagle Medium (DMEM) (Thermo Fisher Scientific, C11995500BT) supplemented with 10% fetal bovine serum (FBS; Thermo Fisher Scientific, A3160801) and 1% penicillin/streptomycin (P/S; Thermo Fisher Scientific, 15140-122) at 37°C in a cultivation chamber with 5% CO_2_. The expression of FOSL1 was inhibited by two oligonucleotide sequences of siRNAs, siRNA-1: 5′-GCUCAUCGCAAGAGUAGCA-3′ and siRNA-2: 5′-GAGCUGCAGUGGAUGGUAC-3′. For siRNA transfection, the cells were planted and transfected with siRNAs by using Lipofectamine RNAiMAX (Life Technologies) according to the manufacturer’s instructions. For the AP-1 inhibitor treatment, the cells were treated with 10 µM T5224 purchased from MedChemExpress (MCE, HY-12270).

### RT-qPCR and western blotting

Total RNA was isolated from cells by using TRIzol reagent (Invitrogen, 15596026) according to the manufacturer’s instructions. For the reverse transcription reaction, cDNA was synthesized using 2 µg RNA with the PrimeScript RT Reagent Kit. RT-qPCR was performed using TB Green Premix Ex Taq II (Takara, RR820A) in a StepOnePlus real-time PCR instrument (Thermo Fisher Scientific). In this study, β-actin served as an internal control. The primer sequences used for RT-qPCR were as follows: FOSL1 (forward):

5′-GCCTGTGCTTGAACCTGA-3′, FOSL1 (reverse): 5′-TGCTGCTACTCTTGCGATG-3′; β-actin (forward): 5′-GATCATTGCTCCTCCTGAGC-3′, β-actin (reverse): 5′-ACTCCTGCTTGCTGATCCAC-3′. For Western blotting, the proteins of the cells were extracted using radioimmunoprecipitation assay (RIPA) lysis buffer (Beyotime, P0013B) according to the manufacturer’s instructions. Then, the proteins were separated by electrophoresis liquid and transferred to a polyvinylidene fluoride (PVDF) membrane. The membrane was blocked with 5% milk for 1 h, incubated with primary antibodies overnight, and then incubated with secondary antibodies for 1 h. The primary antibodies used in this study were FOSL1 (Cell Signaling Technology, #5281) and Glyceraldehyde-3-Phosphate Dehydrogenase (GAPDH) (Proteintech, 10494-1-AP).

### Cell counting kit-8 assay

A Cell Counting Kit-8 (CCK-8) assay (Dojindo, CCK-8-500) was performed according to the manufacturer’s instructions. In brief, 100 μl of the cell suspension containing 1,000 cells was plated on a 96-well plate. The Optical Density 450 (OD450) of each well was measured every 24 h using a microplate reader (Infinite M200 PRO).

### Colony formation

One thousand cells were cultured in six-well plates with 2 ml DMEM supplemented with 10% FBS and 1% P/S for approximately 14 days as described previously (17). The cell culture medium was changed every 2–3 days, and the colony numbers were stained with 0.1% crystal violet for 30 min and calculated using ImageJ.

### Cell migration and invasion assays

Cell migration and invasion assays were performed using 24-well Transwell plates that had an 8-μm pore size (Corning, 3422) and were coated with or without Biocoat Matrigel Matrix (Corning, 354234). Ten thousand cells in 150 μl cell medium without FBS were plated in the upper chambers, and 600 μl medium containing 10% FBS was plated in the lower chambers. After 1 day, all cells were fixed by using 4% paraformaldehyde for 30 min and stained by using 0.1% crystal violet for 30 min. The invaded cells were counted under a microscope.

### Tumor sphere formation assay

One hundred cells were seeded into low-attachment 96-well plates and cultured in DMEM/F12 (Thermo Fisher Scientific, C11330500BT) with 1% B27 supplement (Thermo Fisher Scientific, 12587010), human recombinant Epidermal Growth Factor (EGF) (20 ng/ml; PeproTech, PHG0311), and basic Fibroblast Growth Facto (FGF) (20 ng/ml; PeproTech, 100-18B-10) in an incubator with 5% CO_2_ at 37°C. After approximately 10 days, the number and diameter of spheres were assessed under a microscope.

### Organoid culture

Fresh tumor samples were collected from the Department of Oral and Maxillofacial Surgery, Guanghua School of Stomatology, Sun Yat-sen University. In brief, the samples were digested using Collagenase Type IV (Stemcell, 07909) for 50 min and then filtered through a 100-µm sieve (Falcon, 352360) to remove incompletely digested material. The cell suspension was embedded into the ground substance mixed with DMEM/F12 and Matrigel matrix at a proportion of 1:1. The mixture was seeded into a 24-well plate and cultured in self-configured medium containing DMEM/F12, 1 × B27 supplement, 1 μmol/L prostaglandin E2 (MCE, HY-101952), 10 mmol/L nicotinamide (Sigma, N0636), 1.25 mmol/L N-acetyl-l-cysteine (Sigma, A7250), 50 ng/ml human EFG, 500 nmol/L A83-01 (PeproTech, 9094360), 5 ng/ml human FGF2, 10 ng/ml human FGF 10 (PeproTech, 100-26-5), 0.3 μmol/L CHIR 99021 (Sigma, SML1046), 1 μmol/L forskolin (Abcam, ab120058), 50 ng/ml R-spondin (R&D Systems, 3266-RS), 10 μmol/L Rho-associated kinase (ROCK) inhibitor Y-27632 (TargetMol, T1725), and 25 ng/ml Noggin (PeproTech, 120-10C). The diameter of the organoids was measured after approximately 10 days.

### RNA sequencing analysis and gene expression omnibus dataset analysis

For RNA sequencing (RNA-seq), total RNA was extracted using TRIzol reagent as described above. Total RNA was qualified and quantified using a NanoDrop system and Agilent 2100 bioanalyzer (Thermo Fisher Scientific, MA, USA). Then, mRNA was purified using oligo(dT)-attached magnetic beads. Purified mRNA was fragmented into small pieces at the appropriate temperature by using a fragment buffer. Subsequently, random hexamer-primed reverse transcription was used to produce first-strand cDNA, followed by second-strand cDNA synthesis. Then, according to the manufacturer’s protocols, A-Tailing Mix and RNA Index Adapters were incubated for end repair. The cDNA fragments were amplified by PCR, and the products were purified by Ampure XP beads and then dissolved in EthidiumBromide (EB) solution. For quality control, an Agilent Technologies 2100 bioanalyzer was applied to validate the products. To obtain the final library, the double-stranded PCR products from the previous step were heated, denatured, and circularized by the splint oligo sequence. The single-strand circular DNA was formatted as the final library, which was amplified with phi29 to produce DNA nanoballs that were then used to generate single-end-base reads using the MGIseq500 platform (MGI Technology). To obtain the expression matrix, the sequencing reads were mapped to GRCH38. The related enrichment pathway was performed using gene set enrichment analysis (GSEA) software (http://www.gsea-msigdb.org/gsea/index.jsp). The differentially expressed genes (DEGs) were identified using the DESeq2 R package. The DEGs were identified through a cutoff p-value <0.05 and |log_2_FoldChange| >0.5. Biological function and canonical pathway analyses were performed based on Ingenuity Pathway Analysis (IPA).

In addition, the gene expression microarray of AM from GSE132472 was downloaded, and the samples were divided into two groups according to the average expression of FOSL1. Then, the DEGs with adjusted p-values <0.01 and log_2_FoldChange >1 were identified using the DESeq2 R package. Gene enrichment analysis was performed using the Database for Annotation, Visualization and Integrated Discovery (DAVID) website (https://david.ncifcrf.gov/).

### Statistical analyses

All statistical analyses were performed using GraphPad Prism 9 for Windows (GraphPad Software Inc.). Two-tailed Student’s t-test was performed, and the mean ± SD is shown. A value of p < 0.05 was considered statistically significant.

## Results

### Immunohistochemical expression of FOSL1 in ameloblastomas

**Figure 1:**
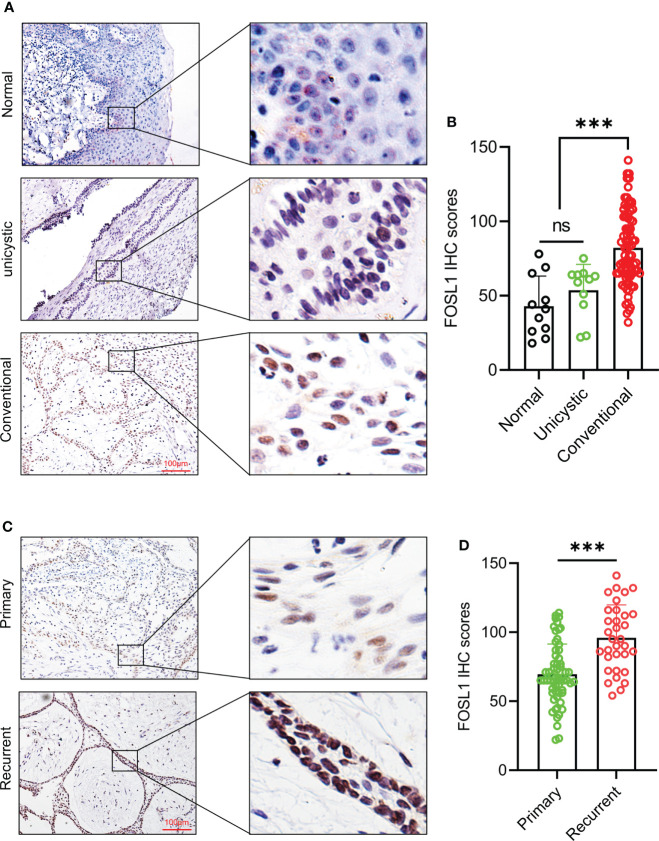
Immunohistochemical expression of FOSL1 in AM **(A, B)** IHC staining images and score quantification of FOSL1 in normal, unicystic, and conventional AMs. Scale bar: 100 μm. ***p < 0.001 by one-way ANOVA. **(C, D)** IHC staining images and score quantification of FOSL1 in primary and recurrent AM. Scale bar: 100 μm. ***p < 0.001 by Student’s t-test. NS, no significance.

To detect the expression and distribution of FOSL1 in AM, we performed an immunohistochemical analysis of 96 AM samples, including 11 unicystic and 85 conventional AM samples. As shown in **Figure 1A**, FOSL1 protein expression can be readily detected in both unicystic and conventional AM. Of note, we observed that the FOSL IHC score was dramatically increased in the conventional AM compared to the unicystic AM and normal oral epithelium (**Figure 1B**). Moreover, compared with the primary AM, the expression of FOSL1 was also enhanced in the recurrent AM (**Figures 1C, D**).

### Knockdown of FOSL1 inhibits cell proliferation, migration, invasion, and tumorosphere formation in ameloblastomas

**Figure 2:**
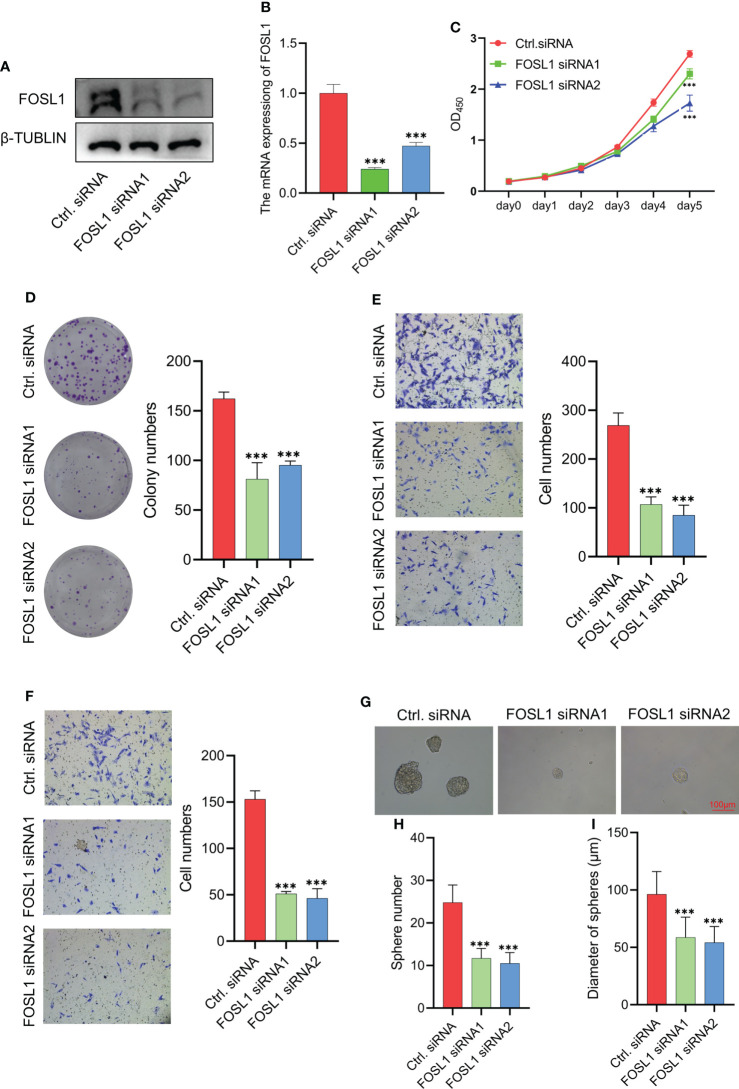
Knockdown of FOSL1 inhibits cell proliferation, migration, invasion, and tumor sphere formation in Ams **(A, B)** Western blotting and RT-qPCR analyses confirmed the knockdown of FOSL1 in AM cells. ***p < 0.001 by one-way ANOVA. **(C–F)** CCK-8 assay **(C)**, colony formation assay **(D)**, migration assay **(E)**, and invasion assay **(F)** were performed on AM cells transfected with control or FOSL1 siRNAs. ***p < 0.001 by two-way ANOVA. **(G–I)** Tumor sphere formation was performed on AMs transfected with the control or FOSL1 siRNAs. ***p < 0.001 by one-way ANOVA.

To clarify the functional role of FOSL1 in AMs, we performed proliferation, Transwell, and sphere formation assays using AMs treated with FOSL1 siRNAs. As shown in **Figures 2A, B**, the expression of FOSL1 protein and mRNA was significantly inhibited in AM cells transfected with FOSL1 siRNAs. The CCK-8 assay showed that depletion of FOSL1 suppressed the proliferation of AM cells (**Figure 2C**). Similar results were also observed in the colony formation assay (**Figure 2D**). In addition, a Transwell assay using plates coated with or without Matrigel showed that cell migration and invasion were reduced significantly in AM cells upon FOSL1 siRNA treatment (**Figures 2E, F**). Of note, we also observed that the number and diameter of AM cell-derived tumorospheres were suppressed in cells with depletion of FOSL1 compared to those of control cells (**Figures 2G–I**).

### Therapeutic target Activating Protein-1 (AP1) suppresses the aggressiveness of ameloblastoma

**Figure 3:**
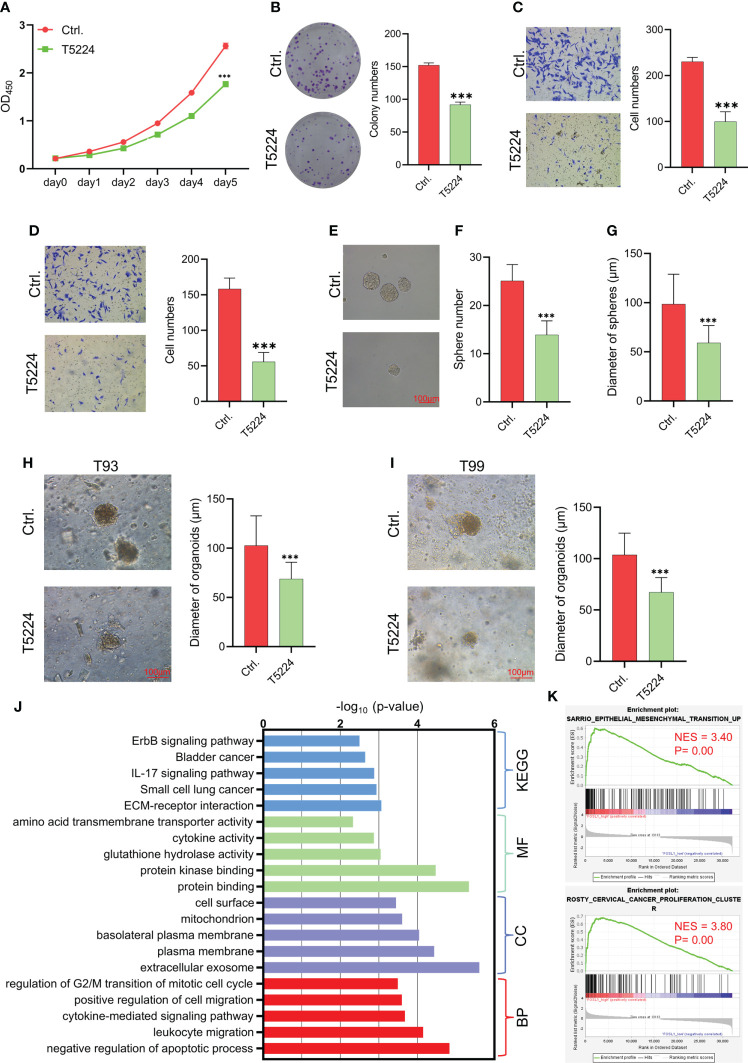
Therapeutic target AP1 suppresses the aggressiveness of AM **(A–D)** CCK-8 assay **(A)**, colony formation assay **(B)**, migration assay **(C)**, and invasion assay **(D)** were performed on AMs with or without T5244. ***p < 0.001 by two-way ANOVA. **(E–G)** Tumor sphere formation was performed on AMs with or without T5244. ***p < 0.001 by Student’s t-test. **(H, I)** Organoids were cultured in T93 and T99 patients with or without T5244. ***p < 0.001 by Student’s t-test. **(J)** GO and KEGG analyses of upregulated DEGs in samples with high FOSL1 expression. The top 5 terms are shown based on the -log_10_(p value). BP, biological process; CC, cellular component; MF, molecular function; KEGG, Kyoto Encyclopedia of Genes and Genomes. **(K)** GSEA showed that cell proliferation- and EMT-associated signatures were highly enriched in samples with high FOSL1 expression. NES, normalized enrichment score.

FOSL1 is a key member of the FOS family of transcription factors that contributes to the formation of the AP1 transcription factor complex. To further evaluate the therapeutic value of targeting AP1 in AM, the AP1 inhibitor T5224 was used to treat AM cells. As expected, T5224 inhibited cell proliferation, migration, invasion, and tumorospheres in AM cells (**Figures 3A** **–** **4G**). Next, we extended these findings in a preclinical patient-derived organoid model. As shown in **Figures 3H, I**, the growth of AM patient-derived organoids was obviously suppressed upon T5224 treatment. To further reinforce our findings in AM samples, the gene expression microarray of AM from GSE132472 was downloaded and analyzed using the DAVID algorithm. A functional enrichment analysis of the Gene Ontology categories biological process (BP), cellular component (CC), and molecular function (MF) and Kyoto Encyclopedia of Genes and Genomes (KEGG) pathways showed that apoptosis, cell cycle, and cell migration were activated in high FOSL1-expressing AMs compared to low FOSL1-expressing AMs (**Figure 3J**). GSEA showed that cell proliferation-related signatures and EMT signatures were also upregulated in FOSL1 high-expressing AMs compared to FOSL1 low-expressing AMs (**Figure 3K**).

**Figure 4:**
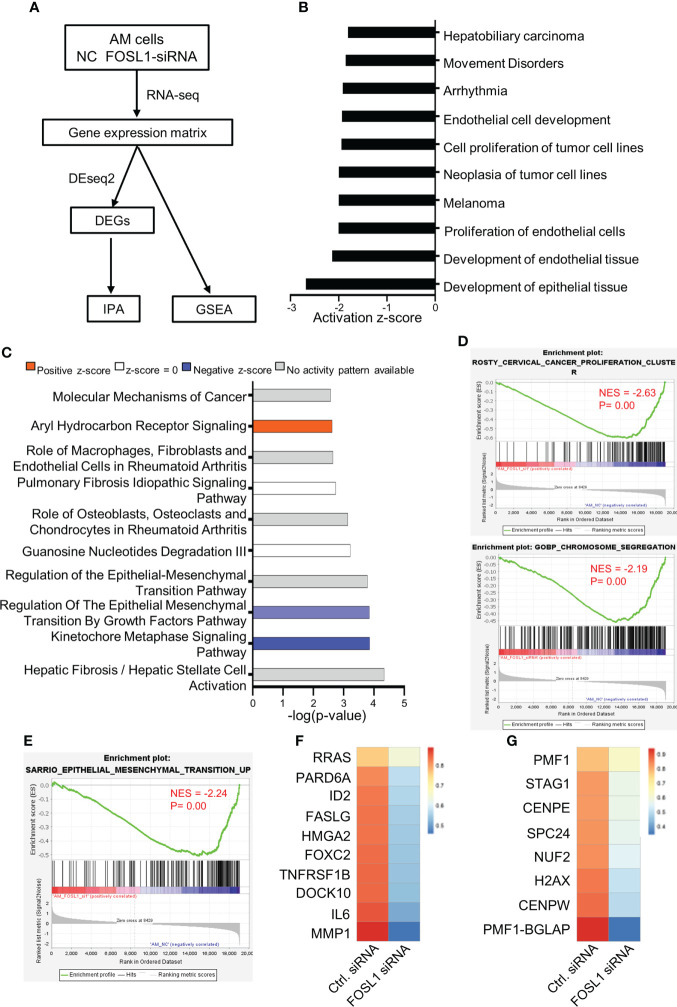
Silencing FOSL1 inhibits kinetochore metaphase signaling and the epithelial–mesenchymal transition pathway in AM **(A)** Flowchart showing the experimental approach of RNA-seq in AM cells. **(B, C)** IPA showed the biological function **(B)** and canonical pathways **(C)** in AMs transfected with EZH2 siRNA. **(D)** GSEA showed that cell proliferation and cell cycle-associated signatures were inactivated in AM cells treated with FOSL1 siRNAs. NES, normalized enrichment score. **(E)** GSEA showed that EMT-associated signatures were downregulated in AM cells treated with FOSL1 siRNAs. NES, normalized enrichment score. **(F, G)** Heatmap showing the gene expression in EMT-associated signatures **(F)** and cell cycle-associated signatures **(G)**.

### Silencing FOSL1 inhibits kinetochore metaphase signaling and the epithelial–mesenchymal transition pathway in ameloblastoma

To further examine the molecular characteristics of AM cells upon FOSL1 depletion, RNA-seq was performed using AM cells with or without FOSL1 knockdown (**Figure 4A**). The biological function analysis showed that the knockdown of FOSL1 mainly affected cell development and proliferation by using IPA (**Figure 4B**). The IPA pathway analysis revealed that kinetochore metaphase signaling and the EMT pathway were inactivated significantly in AM cells that were depleted of FOSL1 (**Figure 4C**). To further confirm these findings, the GSEA also showed that cell proliferation-related signatures and EMT signatures were suppressed in AM cells transfected with FOSL1 siRNAs (**Figures 4D, E**). The heatmap showed that the downregulated genes were associated with cell proliferation and EMT signatures in AM cells upon FOSL1 depletion (**Figures 4F, G**).

## Discussion

**Figure 5:**
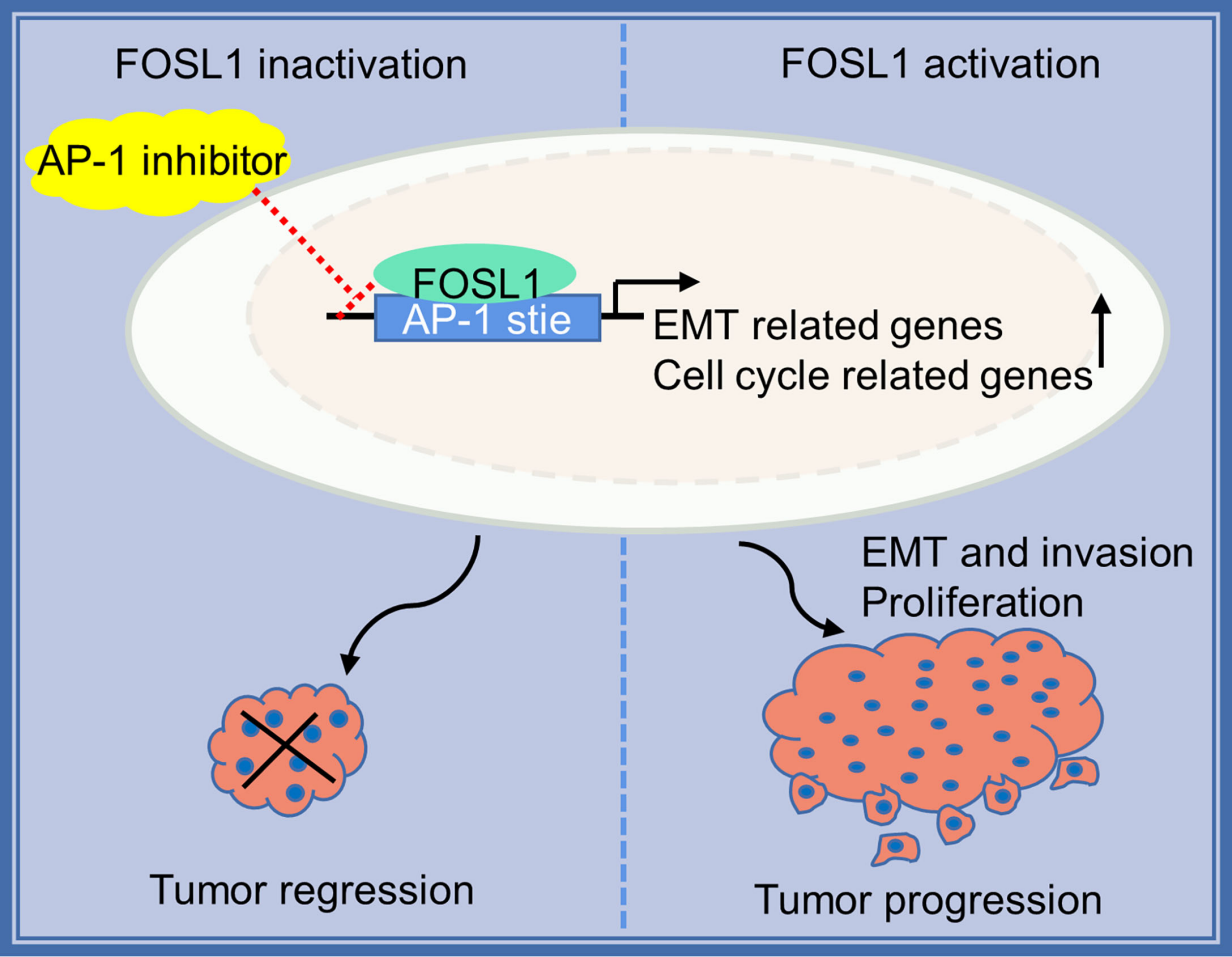
Proposed model for FOSL1 in AM progression.

AM mostly occurs in jaw bones and is the most common odontogenic epithelial tumor, although it is usually benign. However, AMs can also demonstrate local invasive growth and aggressive behavior characterized by marked bone destruction and a high recurrence rate. Increasing evidence has revealed that tumor driver genes or tumor suppressors play important roles in the development and progression of AM (1, 2, 18). Importantly, the BRAF V600 mutation has been confirmed to be the most common and critical oncogenic mutation in AM, and it leads to a continuous overactivation of mitogen-activated protein kinase (MAPK) signaling and its target genes (2, 19, 20). However, the effects of the constitutive expression of these genes are largely unknown. In this study, we showed that FOSL1, a target gene of MAPK signaling (21), was expressed in the nuclei of AM cells and correlated with local tumor invasion and recurrence (**Figure 5**). We further showed that inhibition of FOSL1 suppressed the proliferation and growth of AM cells by targeting key regulators of mitosis, which are involved in kinetochore metaphase signaling. Kinetochores are assembled on centromeric chromatin coordinated with the cell cycle and are essential for accurate chromosome segregation and cell division (22, 23).

Notably, we also observed that depletion of FOSL1 inhibited cell migration, invasion, and sphere formation in AMs. In line with these findings, the IPA and GSEA results showed that the EMT pathway was inactivated in AM cells depleted of FOSL1. The EMT program has a pivotal role in the cancer metastatic cascade, which enables cancer cells to acquire motility, invasiveness, and stemness (15, 24–26). Interestingly, emerging studies have shown that the EMT pathway was activated in AMs and correlated with tumor invasiveness and tumor stem-like cell properties, indicating that EMT might be involved in tumor initiation and local invasion in AMs. Previously, we showed that FOSL1 promoted EMT by activating SNAI2 expression in a superenhancer-dependent manner in head and neck cancer (15, 27), indicating that FOSL1 is a master regulator of EMT-related transcription factors. However, the classic EMT-related transcription factors, including SNAI1, SNAI2, ZEB2, ZEB2, and Twist1, were not obviously changed in AM cells upon FOSL1 depletion. Notably, FOXC2 and HMGA2, which function as transcription factors to induce EMT in solid cancers (28–32), were significantly decreased in AM cells transfected with FOSL1 siRNAs. Interestingly, both FOSL1 and FOXC2 are direct target genes of MAPK signaling (21, 29), which is activated due to the oncogenic BRAF mutation in AM (20, 33). These findings suggested that FOSL1 might promote FOXC2 to induce EMT in AM.

Taken together, our findings revealed that FOSL1 was involved in the invasive growth and recurrence of AM. Genetic deletion and pharmacological inhibition of FOSL1 suppressed cell proliferation, migration, invasion, and sphere formation by inactivating kinetochore metaphase signaling and the EMT pathway. These findings reveal novel therapeutic strategies for AM treatment by targeting FOSL1 or AP-1.

## Data availability statement

The datasets presented in this study can be found in online repositories. RNA-seq data were depositd at the Gene Expression Omnibus (GEO) under access number GSE199107.

## Ethics statement

This study was reviewed and approved by ethical committee of Hospital of Stomatology, Guanghua School of Stomatology, Sun Yat-sen University. Written informed consent for participation was not required for this study in accordance with the national legislation and the institutional requirements.

## Author contributions

CW, GX and OS conceived the study. GX, OS and NX performed most experiments and data analyses. JX, WW, and CY contributed to experiments, data acquisition, and/or analysis. NX performed histopathological review. MZ, XX, DC and CW supervised the study. CW, GX and OS wrote the manuscript, which other authors edited and approved. All authors contributed to the article and approved the submitted version.

## Funding

This work was supported by National Nature Science Foundation of China (82141112) and Guangdong Financial Fund for High-Caliber Hospital Construction (174-2018-XMZC-0001-03-0125/D-14). We thank Prof. Qian Tao from Guanghua School of Stomatology, Sun Yat-sen University for kindly providing hTERT^+^-ameloblastoma cells.

## Conflict of interest

The authors declare that the research was conducted in the absence of any commercial or financial relationships that could be construed as a potential conflict of interest.

## Publisher’s note

All claims expressed in this article are solely those of the authors and do not necessarily represent those of their affiliated organizations, or those of the publisher, the editors and the reviewers. Any product that may be evaluated in this article, or claim that may be made by its manufacturer, is not guaranteed or endorsed by the publisher.
